# Sox10 Is a Specific Biomarker for Neural Crest Stem Cells in Immunohistochemical Staining in Wistar Rats

**DOI:** 10.1155/2020/8893703

**Published:** 2020-08-28

**Authors:** Li-Na Yang, Wen-Kai Huang, Xue-Li Li, Yu-Zuo Bai, Shu-Cheng Zhang

**Affiliations:** ^1^Department of Pediatric Surgery, Shengjing Hospital of China Medical University, Shenyang, 110004, China; ^2^Department of Pediatric Surgery, Wuhan Children's Hospital (Wuhan Maternal and Child Healthcare Hospital), Tongji Medical College, Huazhong University of Science and Technology, Wuhan, 430014, China

## Abstract

**Objective:**

Neural crest stem cells (NCSCs) are prototypically migratory cells immigrating from the dorsal neural tube to specific embryonic sites where they generate a variety of cell types. A lot of biomarkers for NCSCs have been identified. However, which biomarkers are the most specific is still unclear.

**Methods:**

The rat embryos harvested in embryonic day 9 (E9), E9.5, E10, E10.5, E11, E12, E13, and E14 were paraffin-embedded and sectioned in transverse. NCSCs were spatiotemporally demonstrated by immunohistochemical staining with RET, p75NTR, Pax7, and Sox10. NCSCs were isolated, cultured, and stained with RET, p75NTR, Pax7, and Sox10.

**Results:**

In the paraffin sections of rat embryos, the immunohistochemical staining of RET, p75NTR, and Sox10 can all be used in demonstrating NCSCs. Sox10 was positive mainly in NCSCs while RET and p75NTR were positive not only in NCSCs but also in other tissue cells. In primary culture cells, Sox10 was mainly in the nucleus of NCSCs, RET was mainly in the membrane, and p75NTR was positive in cytoplasm and membrane.

**Conclusions:**

Sox10 is the specific marker for immunohistochemical staining of NCSCs in paraffin sections. In cultured cells, Sox10, p75NTR, and RET presented a similar staining effect.

## 1. Introduction

Neural crest stem cells (NCSCs) are prototypical migratory cells of vertebrate embryos; they emigrate from the dorsal neural tube and, by following stereotyped migratory routes, colonize specific embryonic sites where they generate diverse derivatives including the enteric nervous system (ENS) [1]. Therefore, NCSCs are very important for the pathogenesis of intestinal neuronal dysplasia, colonic motility disorders, and functional gastrointestinal diseases [2, 3]. NCSC identification is a key to developmental neurobiology and related disease research. There are a lot of biomarkers for NCSCs. These markers mainly include transcription factor (Sox10, Pax family, Hox family, MASH1, Phox2b, etc.) [4–8], neurotrophic factor receptors (p75NTR, RET, EDNRB, etc.) [7, 9, 10], transgenes (such as D*β*H-lacZ and CCK-lacZ) [11], and various nerve-related proteins such as NF (neurofilament), NC-1, E/C8, HNK-1, nestin, and *α*4-integrin [12–16].

Among the documented markers for NCSCs, neurotrophic factors are the most widely used. Neurotrophins are growth factors that play critical roles in the development, maintenance, survival, and death of NCSCs. RET is a member of the receptor tyrosine kinase (RTK) superfamily, which is the major HSCR gene [17, 18], and RET signaling activity is crucial for vagal neural crest development [19]. Ding et al. used RET to mark NCSCs in ganglia and analyzed the distribution of the CXCR4 expression in different colon segments from HSCR patients [20]. p75NTR is a low-affinity receptor of neurotrophic factor and has been identified as a member of the TNF receptor superfamily that now contains about 25 receptors, including tumor necrosis factor receptor 1 (TNFR1) and TNFR2, Fas, RANK, and CD40. Neural crest cells express p75NTR as they migrate and accumulate at sites of formation of sensory and sympathetic ganglia, and levels increase substantially in cells of these ganglia as they aggregate and undergo neuronal differentiation [21]. Isolation by p75NTR expression is a widely used tool for NCSC purification [22–24].

Among the transcription factors, Pax3 and Pax7 have been identified as important players in the genetic network of NC specification [25]. Jiao et al. executed immunofluorescent staining of HNK-1, Pax7, and Ap-2*α* demonstrating that cranial NCSC generation was inhibited by okadaic acid exposure [26]. Sox10 is a member of the SOX family of transcription factors that is expressed in delaminating neural crest cells and is essential for proper ENS development [27]. Hirst et al. carried out immunohistochemistry to examine whether the colonization of the gut by enteric NCSCs is delayed in embryonic Kif1bp−/− mice using the neural crest cell marker, Sox10, and the distance from the ileocaecal junction to the most caudal Sox10+ cell measured [28].

All of the above four families represent the most popular biomarkers for NCSCs because of their roles in NCSC migration and proliferation. However, it is not yet clear which marker is more suitable for demonstrating NCSCs in immunological staining. Therefore, we selected RET, p75NTR, Pax7, and Sox10 as the targets to screen the most suitable markers for NCSCs in immunological staining in tissue sections of normal rat embryos and NCSC culture from rat, which, in an almost definite way, could provide clues for the application of similar methods related to observation of NCSCs in the future.

## 2. Materials and Methods

### 2.1. Animal Model and Tissue Collection

This study was approved by the Institutional Ethics Committee of China Medical University Shengjing Hospital. All of these experiments in the current research were in compliance with the government policies and defined protocols. Mature Wistar rats (body weight: 250 to 300 g) were provided by the Medical Animal Center, Shengjing Hospital of China Medical University (Shenyang, China). The male and female rats were caged at midnight at a ratio of 1 : 4. The vaginal smears of the female rats were made at 8:00-9:00 a.m. the next day. The day at which a vaginal plug was detected was designated embryonic day 0 (E0). The rat embryos were obtained by cesarean delivery in E9, E9.5, E10, E10.5, E11, E12, E13, and E14. They were fixed in 4% paraformaldehyde for 12 to 24 h, depending on their size, and then dehydrated, paraffin-embedded, and serially sectioned in transverse at a thickness of 3.5 *μ*m preparing for immunohistochemical staining.

### 2.2. Isolation and Culture of NCSCs

NCSCs were isolated from the embryonic site of E12 Wistar rats. The abdomen containing the gut was dissected and then mechanically and enzymatically dissociated with 0.2% collagenase IV (1 mg/mL; Sigma Aldrich, USA) at 37°C for 30 min with gentle vibration. Cells were filtered through a 40 *μ*m cell strainer to obtain a single suspension, which was cultured at 500,000 cells/mL in DMEM/F12 basic medium (Invitrogen, USA) supplemented with 2% B27 (Invitrogen, USA), 20 ng/mL epidermal growth factor (EGF, Peprotech, USA), 10 ng/mL basic fibroblast growth factor (bFGF, Peprotech, USA), 0.0002% heparin, and 100 U/mL penicillin-streptomycin (Invitrogen, USA) for 3-4 days to form neurospheres. Neurospheres were passaged every 5–6 days with Accutase (Gibco, USA) at 37°C for 30 minutes and centrifuged at 480g for 5 min to encourage cell aggregation. Cell suspension was replated at 100,000 cells/mL in a 6-well round bottom plate (Corning, USA), which was coated with 0.01% collagen from the rat tail (Sigma, USA). NCSCs at passage 2 were then resuspended and had replating in preparation for immunocytochemical staining.

### 2.3. Immunohistochemical and Immunocytochemical Staining

The immunohistochemical staining experiment uses the S-P method. The specific experimental steps refer to the kit (MXB Kit-9710, CHN) instructions. After the improvement according to the experimental conditions, the approximate operation steps are as follows: Antigen retrieval was performed by heating the sections in 10 mmol/L citrate buffer (pH 6.0) at 98°C for 5 min. Sections were blocked by incubation with endogenous peroxidase blockers (Reagent 1) for 20 min at room temperature (RT) and animal nonimmune serum (Reagent 2) for 30 min at RT. Then, following overnight incubation at 4°C in primary antibody (Pax7 (1 : 100, Santa Cruz, USA), p75NTR (1 : 100, Proteintech, USA), RET (1 : 100, Abcam, UK), and Sox10 (1 : 100, Santa)), sections were incubated in horseradish peroxidase- (HRP-) conjugated secondary antibody (Reagent 3) and streptomyces antibiotic protein-peroxidase (Reagent 4) each for 10 min at RT. Signals were visualized using 3,3′-diaminobenzidine. Sections were counterstained with hematoxylin. Finally, sections were mounted with neutral balsam. Sterile round cover slides with cells were used for immunocytochemical staining. After fixing in 4% paraformaldehyde for 20 min, slides were treated with 0.2% Triton X-100 for 5 min. The rest of the operations were according to the kit instructions (Pax7 (1 : 100, Santa Crus, USA), p75NTR (1 : 150, Proteintech, USA), RET (1 : 100, Abcam, UK), and Sox10 (1 : 100, Santa)).

### 2.4. Image Acquisition

After observation under a fluorescence microscope (Olympus Microscope, LLC, Dushinjuku, TKY) and photography on the Nikon E800 imaging system, 5 target areas (where NCSCs are located) and 5 nontarget areas (excluding NCSCs) were randomly selected. Image brightness and contrast were adjusted using Photoshop CC (Version 14.0, Adobe Systems, Inc., San Jose, CA).

## 3. Results and Discussion

### 3.1. Immunohistochemical Staining of Sox10, Pax7, p75NTR, and RET for NCSCs in Paraffin Sections

**Figure 1:**
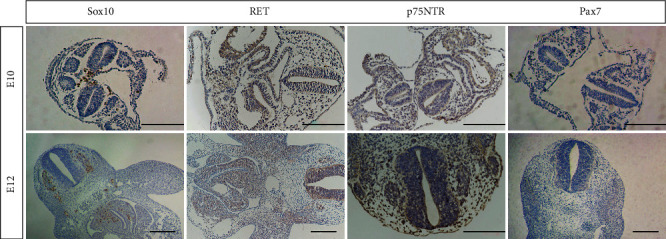
Comparison of immunohistochemical staining results for Sox10, Pax7, p75NTR, and RET in normal rat embryos of different developmental stages (E10 and E12). Scale bar, 100 *μ*m.

Immunohistochemical staining of NCSCs labeled by Sox10, Pax7, p75NTR, and RET was carried out in tissue sections of different developmental stages (E9, E9.5, E10, E10.5, E11, E12, E13, and E14). The results showed that Sox10 was the most significant in the rat embryo staining of E10 and E12; the specificity was very high indicating that Sox10 was the preferred marker of NCSCs. Sox10 was mainly expressed in the nucleus of NCSCs, while RET, p75NTR, and Pax7 also expressed strongly in NCSCs, but in addition to expression in NCSCs, they also expressed in mesenchymal cells, stromal cells, the thyroid or salivary gland precursor cells, or other precursor cells (Figure 1). Based on the above results, we determined that Sox10 was the most specific morphological marker for NCSC immunological staining in paraffin sections.

### 3.2. The Migratory Pathway of NCSCs in Rat Embryos Demonstrated by Sox10 Staining

**Figure 2:**
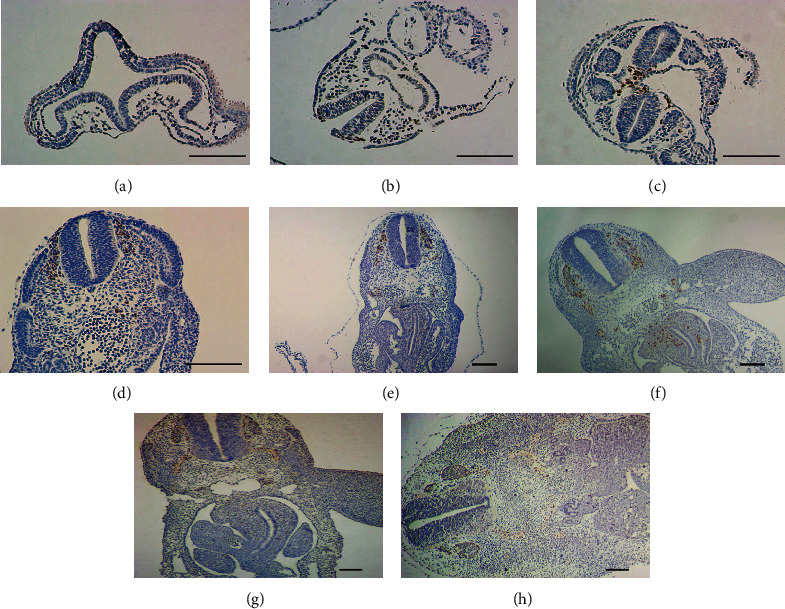
The migratory pathway and pattern of NCSCs were observed with Sox10 as a marker. (a) E9, the neural tube has not been completely closed, and the neural crest has not been formed. (b) E9.5, the neural crest began to form, and NCSCs were delaminated. (c) E10, NCSCs migrated symmetrically to both sides along the gap between neural tube and epidermis. (d) E10.5, NCSCs migrated to both sides of salivary glands and chordates. (e) E11, NCSCs migrated to both sides of the dorsal aorta, and some cells entered the bilateral pericardial cavity. (f) E12, NCSCs were transplanted in the pericardial cavity, and some cells began to pass through the original diaphragm and enter the anterior intestine, middle intestine, pancreas, and spleen. (g) E13, NCSCs began to colonize the intestinal wall. (h) E14, ENS is formed. Scale bar, 100 *μ*m.

Based on the above results, we chose Sox10 as the marker of NCSCs, and the migratory process of NCSCs was observed by immunohistochemical staining. In E9.5 sections, the neural crest was formed from where NCSCs began to be delaminated. In E10 sections, NCSCs migrated sideways along the bilateral gap between neural tube and epidermis. In E10.5 sections, NCSCs migrated to the head and neck and on both sides of the salivary glands and spinal cord. In E11 sections, NCSCs arrived at both sides of the dorsal aorta and some cells entered the bilateral pericardial cavity. In E12 sections, NCSCs were transplanted in the pericardial cavity, and some cells began to pass through the original diaphragm and entered the anterior intestine, middle intestine, pancreas, and spleen. In E13 sections, NCSCs began to colonize and differentiate in the muscles of the intestinal wall. Finally, ENS was formed in E14 (Figure 2).

### 3.3. Immunocytochemical Staining of Sox10, Pax7, p75NTR, and RET in Cultured NCSCs

**Figure 3:**
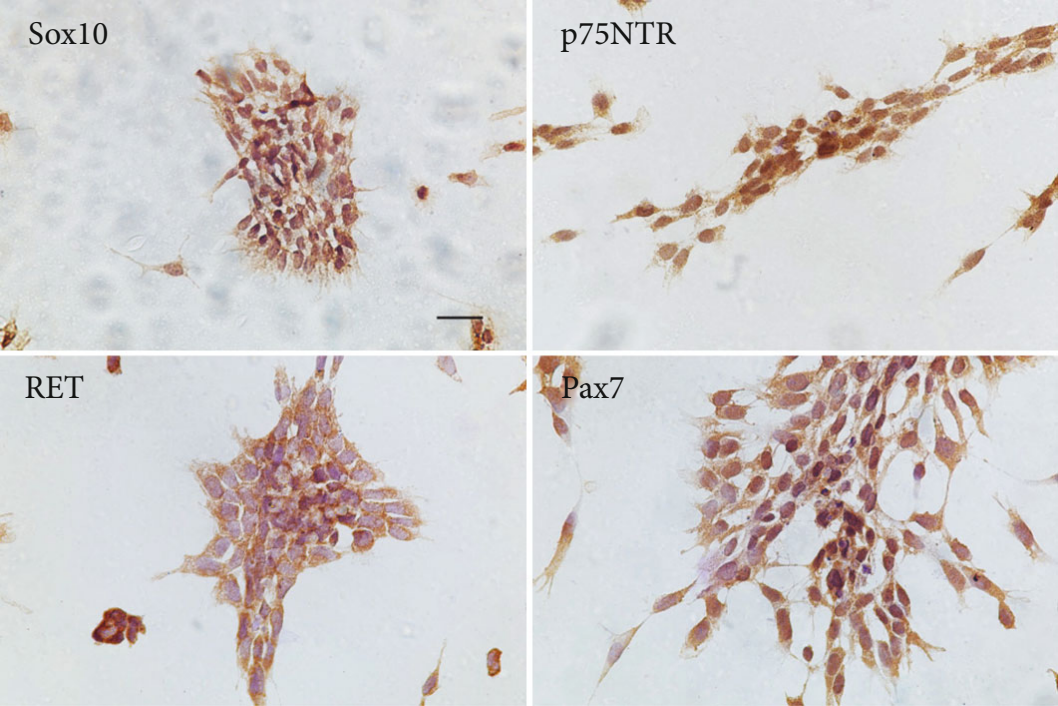
Immunological staining for Sox10, Pax7, p75NTR, and RET in primary cell culture of ENCCs isolated from E12 normal rat embryos. Scale bar, 50 *μ*m.

In view of the fact that Sox10 was most expressed in the stage of E10 and E12, we selected E12 rat embryos to isolate NCSCs for primary culture and immunocytochemical staining. NCSCs identified by Sox10, Pax7, p75NTR, and RET showed that Sox10 was mainly positive in the nucleus of NCSCs, RET was mainly located at cytomembrane, p75NTR was positive in the cytoplasm and membrane, and Pax7 was in the cell nucleus and cytoplasm (Figure 3). From the perspective of cell morphology, the nucleus of NCSCs was larger than that of ordinary cells, occupying most of the volume of the cell body, which made the markers expressing on the nucleus more dominant in the results of immunocytochemical staining. p75NTR, Sox10, and RET had similar staining effect. However, the staining results of Pax7 were not uniform. Some cells do not express Pax7. Based on the above results, we determined that Sox10, p75NTR, and RET are sensitive morphological markers for NCSC immunological staining in primary cell culture.

## 4. Conclusions

NCSCs are typically identified by expression of genes thought to regulate specific aspects of NCSC fate. These markers include Sox10, p75NTR, Ret, and Pax7. It is necessary to select a suitable marker to identify NCSCs. This study investigated the four markers labeling NCSCs by immunohistochemistry and immunocytochemistry technology. As a result, Sox10 was the most specific marker for NCSCs in tissue sections, and Sox10, p75NTR, and RET had a similar staining effect in cultured cells.

Sox10 is a transcription factor with a central role in the late stages of differentiation of the neural crest, peripheral nervous system, and melanocytic cells in humans [29–31]. The vagal neural crest cells can express Sox10 when they move out of the neural tube, continue to express during the NCSC migration, and maintain the activity of ENS precursor cells. Sham et al. studied Sox10 expression in Hirschsprung's intestine and found that Sox10 was expressed in neurons and glial cells in the normal intestinal neuroplexus, whereas the expression of Sox10 in the nonganglionic gut was decreased [32]. It indicates that maintaining appropriate levels of Sox10 expression is critical for the development and function of the ENS. Animal experimental studies have confirmed that the development of the enteric nervous system in mice lacking Sox10 has been arrested [27, 33].

Sox10 labeling NCSCs are excellent in immunological staining as a result of some reasons. Firstly, in terms of immunoreactive specificity, the immunohistochemical results of tissue sections showed that Sox10 was almost exclusively expressed in NCSCs and was rarely expressed in other cells. While the other three markers (p75NTR, Pax7, and RET) were expressed not only in NCSCs but also in other cells, they are not specific. RET and p75NTR were widely expressed in other cells such as mesenchymal cells, stromal cells, the thyroid or salivary gland precursor cells, or other precursor cells, which is far less specific to labeled NCSCs than Sox10. Secondly, Sox10, as a key transcription factor, was significantly expressed on the nucleus of NCSCs and was rarely expressed in cytoplasm and cell membranes. By morphological observation, we found that NCSCs, as a type of stem cells, had all the common characteristics of stem cells. That is, the nucleus of NCSCs was so large that it was proportioned about four-fifths of the cell body, while the cytoplasm and cell membrane together only accounted for one-fifth of the entire cell. However, as a neurotrophic factor receptor, RET was mainly located in the cell membrane, which objectively determined that its staining effect was not as good as Sox10. Therefore, Sox10 is the most prominent and specific marker in screening NCSCs of tissue sections by immunohistochemistry.

Although Sox10 is apparent in tissue sections, it has no advantage in cell immunostaining to identifying NCSCs. After immunocytochemical staining, it was found that Sox10, p75NTR, and RET were all satisfactory. As a neurotrophic factor receptor, p75NTR was expressed in the cytoplasm and cell membrane. The use of p75NTR appears to be more suitable and is currently applied most widely. Intriguingly, p75NTR expression is enriched at a number of mesenchymal/epithelial boundaries. Mesenchymal cells located within several developing organs (limb, kidney, maxillary pad, tooth, lung, muscle, testis, retina, and pituitary) express p75NTR at very high levels, and p75NTR is also abundant in the mesenchyme surrounding developing epithelial structures. This is why p75NTR is not an ideal marker for NCSCs in immunological staining of sections but for cultured cells. RET is one of the most widely recognized NCSC markers. The overall staining effect of RET is similar to that of p75NTR in immunocytochemical staining, which may be the reason for the amount of expression. But p75NTR is better in early migrated NCSC; RET is better in late. Pax7 is a transcription factor expressed in the nucleus like Sox10; Pax7 labeled not only NCSCs but also the anteroposterior axis and abdominal distension area of the embryo. The results from our study suggest that Pax7 was widely expressed around the entire neural tube subcutaneously and was distributed symmetrically along the anterior-posterior axis of the embryonic body. This is also consistent with the expression of Pax7 during mouse embryonic development. The expression of Pax7 was not uniform in cell culture. Pax7 showed significant staining in the nuclei of some cells and in the cytoplasm of others. Even so, some cells do not express Pax7.

According to our study, Sox10 is the best marker for NCSCs. This provides a reliable molecular marker for future research on neural crest development. For example, migration pathways associated with the development of NCSCs can be observed through immunohistochemical staining by Sox10. Herein, we demonstrated that the development of ENS originates from the vagal neural crest and the migratory pathway of NCSCs from the neural crest to the intestinal tract and colonization was clearly shown (see the results). In addition, Sox10 staining can also be used in other areas of NCSCs, such as neural tracing or NCSC transplantation. Currently, p75NTR is commonly used to mark NCSCs in relevant transplantation treatment studies [34, 35]. However, p75NTR is not only expressed in NCSCs but also continuously expressed in neurons derived from NCSC differentiation, which has a certain degree of interference to various assessments. Sox10 is expressed in the early migrating neural crest cell population, which is proliferative and undifferentiated [36]. Therefore, Sox10 may be one of the required markers. In a word, it has a broad application prospect and is recommended.

This study had several limitations. Firstly, we only verified the reliability of Sox10 in rats but did not verify it in other model organisms, such as mice, zebrafish, or chickens. Whether Sox10 has species specificity is not clear, and more model biological data will be needed to verify it in the future. On the other hand, we just verified neurotrophic factor receptors and transcription factors to identify NCSCs, which are typically used. The staining effect of other infrequently used markers, such as transgenes and nerve-related proteins, remains to be elucidated.

In conclusion, Sox10 is the most sensitive marker for immunohistochemical staining of NCSCs in paraffin sections obtained from Wistar rats.

## Data Availability

The data used to support the findings of this study are included within the article.
